# After-War Developments for the Voluntary Hospital

**Published:** 1919-11-29

**Authors:** 


### AFTER-WAR DEVELOPMENTS FOR THE VOLUNTARY HOSPITAL.

THE resignation of Sir Cooper Perry of the Vice-Chancellorship of London University, and of the superintendentship of Guy's Hospital (see page 181), may prove momentous in the history of the voluntary hospital system throughout Great Britain. In order to make the position plain and, as we hope, to facilitate extensive preparations throughout the country which are called for if Great Britain is wise enough to lead the world in hospital reorganisation —seeing that the field is open to the nation to-day— an exposition of the present position, in the existing special circumstances of the moment, may make Great Britain's policy a basis for the reconstruction of the hospital system of many countries throughout the world. It is our purpose in these articles to endeavour to arouse hospital and public opinion throughout the country to the important and pressing urgency for the reorganisation of the voluntary hospital system, and the extension of the work and the methods of the work done by or through this system. The lessons taught by the individual and great hospitals, their past history, difficulties, developments, extensions, and present position, must be mastered and understood. A knowledge of the special benefits and protection which this system has afforded and does afford to every individual, whatever their rank or position, who enters one of this group of hospitals as a patient for treatment, is in fact vital to the best interests not only of the patients under treatment, but of all classes of the community. The voluntary hospital system is the only system in the world to-day which puts the claims and Christ-like treatment of every patient in a hospital before every other consideration. The present upheaval and disturbed condition of public opinion, and the lamentable incompleteness of the knowledge of vital facts which relate to it, have given rise to the gravest misapprehensions on the part of labour and the artisan classes. If this continues unremoved, and the true conditions of hospital treatment under other systems are not brought to the mind and understanding of those who are labouring under a deception of view, the consequences may be lamentable. If the hospitals in this country were to be put upon the rates, as many are thoughtlessly advocating at the moment, the future well-being of the people, their treatment, nursing, and the conditions under which both are carried on, would be materially affected for the worse; whilst the way would be opened for dangerous innovations, which the working classes might have to suffer under, to their own undoing and that of those members of their families who had recourse to these institutions.

Such must be the result should the day ever come when Florence Nightingale's teaching and example, and the cardinal principles in voluntary hospital administration, are disregarded. These principles are that the patient's interests and safety shall be always, as they are under the voluntary system, enforced in all hospitals before every other consideration whatever. It is the duty of everyone who aspires to be a leader of the working and artisan classes to obtain a copy of THE HOSPITAL for September 27 last, and to read and preserve the article on pages 633-634 of that number, which explains why the voluntary hospital is a bulwark of security for the patient; the consequences of an evil discipline; the position of the patient in hospitals under the voluntary system; the responsibility of silence; why the maintenance of voluntary hospitals ministers to public safety, and the best interests of every sick patient within hospital walls; foreign government and municipal hospitals; why English men and women would never rest happy to be patients in the general wards of foreign Government and municipal hospitals; a wise and useful reminder that the maintenance of the voluntary hospital system is essential to the well-being and safety of all classes; why to be a Briton, an English man, Woman or Child is indeed a glorious privilege, a great honour, a grave responsibility and our noblest heritage, guaranteeing as the right of every one of us the maximum security and the tenderest and most considerate treatment in a voluntary hospital when suffering from accident, or overtaken by illness or injury which may necessitate operative or other grave risks, in an attempt to prolong life or preserve the continuous blessings of recovered health. These are the birthrights of every free man, woman and child in our midst.

(NOTE.—The Scientific Press, 28-29 Southampton Street, Strand, W.C., is publishing a pamphlet, which will be ready shortly, entitled "The Voluntary Hospital Essential to Public Safety." Every man and woman of intelligence, who takes an interest in their own well-being and security, should make it their business to procure a copy of this small publication, which should find a place in every hospital throughout the land. The price will be within the compass of all.)

### A WONDERFUL PIECE OF HOSPITAL ACHIEVEMENT.

### Guy's in Peril.

IN 1893 it was becoming increasingly apparent to those who had the best interests of Guy's Hospital at heart that, owing to the considerable depreciation in land values and the diminished income from such properties, as well as from other causes, the finances of Guy's Hospital were assuming a perilous condition of inadequacy and danger. This state of affairs was becoming recognised, and three years later, in 1896, His Royal Highness the Prince of Wales (afterwards King Edward VII.) was invited to preside at a public banquet to be held in the summer. King Edward, as he evidenced, had become personally interested in the position of the London hospitals, and alive to the huge sums

**After-War Developments for the Voluntary Hospital** —(*continued*).

which would be necessary to enable the elder and more ancient of their number to rebuild, reconstruct, or take other serious and drastic steps to modernise the buildings of these institutions, and to replenish the exchequers of several of them. At that time it was thought a remarkable feat to raise as much as £10,000 from a banquet. The Prince of Wales realised how perilous the position of Guy's Hospital was in fact, and that nothing but extraordinary and sustained efforts could hope to make its hygienic and structural condition satisfactory and up to date. With a wisdom which often characterised his decisions in the later years of his life he boldly established a new precedent by declining the invitation to preside at the Guy's Hospital dinner. This act fell as a bomb-shell amongst those immediately responsible for the affairs of the hospital. Meetings were called and consultations held, which speedily resulted in the awakening of everybody who had the interests of Guy's Hospital at heart to the pressing need which existed for the remaking and reconstruction of Guy's Hospital.

Briefly the position was found to be one where a sum of £500,000 must be raised and added to the Endowment Funds of the hospital, and £300,000 to £500,000 more provided for the buildings, necessary extensions, reconstruction, replacements, and new structures so seriously needed to secure a fully-equipped, up-to-date and hygienically perfect hospital on the existing Guy's Hospital site. Dr. Cooper Perry, when he took up the superintendentship at Guy's at the end of 1893, after studying the position and having an opportunity of going fully into the whole of the Guy's Hospital problem and mastering it, was fully seized of the facts when the Prince's bomb fell amongst the Governors and responsible managers of this hospital. Within two months of the Prince of Wales's refusal important decisions were arrived at. Dr. Perry and Mr. (now Sir) Alfred Fripp were appointed honorary secretaries of the Re-endowment Fund, and Mr. Cosmo Bonsor, D.L., accepted the office of Treasurer. Numerous committees were appointed, and everybody associated or having any connection with Guy's Hospital came forward and offered their services in support of a splendid effort to put Guy's Hospital on its feet, give it the necessary financial strength and secure its conversion into a modern, up-to-date hospital of the best.

When the Prince of Wales learnt what was happening he consented to preside at a great festival dinner to be held at the Imperial Institute under remarkable auspices, with the result that in less than two months this united action resulted in the collection of £167,528, which sum was received in response to the appeal of the Prince of Wales and announced during the dinner. The actual capital value of the sum forthcoming, when the annual subscriptions and additional revenue obtained were capitalised, amounted in round figures to some £360,000. One of the most remarkable features of this collection was the sum raised through the instrumentality of the medical staff and the members of the medical profession throughout the country. The Prince of Wales in his address stated that of the many classes who had generously responded to his appeal few if any had contributed more liberally, in accordance with their means, than the former graduates of Guy's, who in all parts of the world actively co-operated with their late teacher and friend Dr. (afterwards Sir) Samuel Wilks, President of the Royal College of Physicians. The task devolving upon the honorary secretaries, Dr. Cooper Perry and Mr. Alfred Fripp, was of course onerous, but the services in this connection of the new treasurer, Mr. Cosmo Bonsor, who is the Present President of the hospital, were herculean and extraordinarily fruitful, not only in connection with the Prince's appeal but continuously for the space of several years. The Prince of Wales stated at the dinner that the sum necessary adequately to strengthen the endowments of Guy's Hospital was half a million of money, that is to say, an increased income of £15,000 a year, which if capitalised would represent the capital sum required. Mr. Cosmo Bonsor, the President of Guy's Hospital to-day, is in the proud position of knowing that not only has the desired sum for re-endowment been practically obtained in cash, but that considerably more than that sum has been raised if the additions to revenue which have resulted are capitalised, and some £400,000 more for reconstruction work added.

Here is exhibited Sir Cooper Perry's wonderful grasp of administrative detail, architectural, structural, and technical, with other essentials of knowledge requisite to the successful mastery of all the difficulties and requirements engendered by the reconstruction of great masses of buildings like those of Guy's. He set himself to master all these, and produced what is to-day substantially a new Guy's Hospital, up to date and marvellously efficient in practically every detail. In order to make his task successful under the specially difficult conditions which prevailed at Guy's Hospital in regard to its site, the conditions of the old buildings, modern hospital requirements, and the essential need of success in accomplishment, wherever reconstruction was undertaken, under Sir Cooper Perry's direction and supervision, the whole of these works have been carried out by the hospital's own staff of workmen and technical assistants, with the co-operation and aid of the hospital surveyor, who have cordially co-operated with Sir Cooper Perry in the rebuilding, reconstruction and completion of this vast undertaking. They have brought it already to a state of completion and efficiency which has won the admiration and recognition of expert hospital authorities, as well as of the medical, surgical, nursing, and indeed all members of the staff who are responsible for the conduct of the daily routine and continuous work connected with the treatment of the patients and everything requisite to secure their speedy recovery and continuous well-being within the hospital walls.

Speaking as one who has spent a lifetime in work amongst the hospitals of all types, and is acquainted through personal inspection with all the great hospitals in the world, with few exceptions, we can conscientiously add our tribute to the eulogiums and gratitude which have been universally expressed to Mr. Cosmo Bonsor, Sir Cooper Perry,

(*Continued on p.* 191.)

### After-War Developments for the Voluntary Hospital

(*continued from p.* 190.)

the surveyor and his staff, and all who have had any hand in attaining the magnificent results to which we here call attention. Our space is exhausted, and we must hold over till next week an exposition of the after-war developments for the voluntary hospital. In regard to these the people in every centre of population where hospitals are to be found in Great Britain, following the example of Guy's Hospital, must brace their loins and prepare themselves to help to set these After-War Developments, well going within the next three years.

(*To be continued.*)

